# Drug adherence and multidisciplinary care in patients with multiple sclerosis: Protocol of a prospective, web-based, patient-centred, nation-wide, Dutch cohort study in glatiramer acetate treated patients (CAIR study)

**DOI:** 10.1186/1471-2377-11-40

**Published:** 2011-03-30

**Authors:** Peter J Jongen, Gerald Hengstman, Raymond Hupperts, Hans Schrijver, Job Gilhuis, Joseph H Vliegen, Erwin Hoogervorst, Marc van Huizen, Eric van Munster, Johnny Samijn, Els de Schryver, Theodora Siepman, Martijn Tonk, Eveline Zandbergen, Jacques ten Holter, Ruud van der Kruijk, George Borm

**Affiliations:** 1 MS4 Research Institute, Nijmegen, the Netherlands; 2 Catharina Ziekenhuis, Eindhoven, the Netherlands; 3 Academic MS Centre Limburg, Orbis Medical Centre, Sittard, the Netherlands; 4 Westfries Gasthuis, Hoorn, the Netherlands; 5 Reinier de Graaf Gasthuis, Delft, the Netherlands; 6 Medisch Spectrum Twente, Enschede, the Netherlands; 7 St. Antonius Ziekenhuis, Nieuwegein, the Netherlands; 8 Scheper Ziekenhuis, Emmen, the Netherlands; 9 Jeroen Bosch Ziekenhuis, Den Bosch, the Netherlands; 10 Maasstad Ziekenhuis, Rotterdam, the Netherlands; 11 Rijnland Ziekenhuis, Leiderdorp, the Netherlands; 12 Erasmus Medisch Centrum, Rotterdam, the Netherlands; 13 Leids Universitair Medisch Centrum, Leiden, the Netherlands; 14 Rijnstate Ziekenhuis, Arnhem, the Netherlands; 15 Deventer Ziekenhuis, Deventer, the Netherlands; 16 Slingeland Ziekenhuis, Doetinchem, the Netherlands; 17 Department of Epidemiology, Biostatistics and Health Technology Assessment, Radboud University Nijmegen Medical Centre, Nijmegen, the Netherlands

## Abstract

**Background:**

Multiple sclerosis (MS) is a chronic inflammatory demyelinating disease of the central nervous system, for which no definitive treatment is available. Most patients start with a relapsing-remitting course (RRMS). Disease-modifying drugs (DMDs) reduce relapses and disability progression. First line DMDs include glatiramer acetate (GA), interferon-beta (INFb)-1a and INFb-1b, which are all administered via injections. Effectiveness of DMD treatment depends on adequate adherence, meaning year-long continuation of injections with a minimum of missed doses. In real-life practice DMD-treated patients miss 30% of doses. The 6-month discontinuation rate is up to 27% and most patients who discontinue do so in the first 12 months.

Treatment adherence is influenced by the socio-economic situation, health care and caregivers, disease, treatment and patient characteristics. Only a few studies have dealt with adherence-related factors in DMD-treated patients. Self-efficacy expectations were found to be related to GA adherence. Patient education and optimal support improve adherence in general. Knowledge of the aspects of care that significantly relate to adherence could lead to adherence-improving measures. Moreover, identification of patients at risk of inadequate adherence could lead to more efficient care.

In the near future new drugs will become available for RRMS. Detailed knowledge on factors prognostic of adherence and on care aspects that are associated with adequate adherence will improve the chances of these drugs becoming effective treatments. We investigate in RRMS patients the relationship between drug adherence and multidisciplinary care, as well as factors associated with adherence. Given the differences in the frequency of administration and in the side effects between the DMDs we decided to study patients treated with the same DMD, GA.

**Methods/design:**

The Correlative analyses of Adherence In Relapsing remitting MS (CAIR) study is an investigator-initiated, prospective, web-based, patient-centred, nation-wide cohort study in the Netherlands.

The primary objective is to investigate whether GA adherence is associated with specific disciplines of care or quantities of specific care. The secondary objective is to investigate whether GA adherence is associated with specific aspects of the socio-economic situation, health care and caregivers, disease, treatment or patient characteristics.

All data are acquired on-line via a study website. All RRMS patients in the Netherlands starting GA treatment are eligible. Patients are informed by neurologists, nurses, and websites from national MS patient organisations. All data, except on disability, are obtained by patient self-reports on pre-defined and random time points. The number of missed doses and the number of patients having discontinued GA treatment at 6 and 12 months are measures of adherence. Per care discipline the number of sessions and the total duration of care are measures of received care. The full spectrum of non-experimental care that is available in the Netherlands is assessed. Care includes 'physical' contacts, contacts by telephone or internet, health-promoting activities and community care activities. Care received over the preceding 14 days is assessed by patients at baseline and every other week thereafter up to month 12. Every 3 months neurologists and nurses record care disciplines to which patients have been referred.

The Dutch Adherence Questionnaire-90 (DAQ-90) is a 90-item questionnaire based on the World Health Organisation (WHO) 2003 report on adherence and comprehensively assesses five domains of evidence-based determinants of adherence: socio-economic, health care and caregivers, disease, treatment, and patient-related factors. In addition, self-efficacy is assessed by the MS Self-Efficacy Scale (MSSES), and mood and health-related quality of life (HRQoL) by the Multiple Sclerosis Quality of Life-54 questionnaire (MSQoL-54). Relapses and adverse events probably or definitively related to GA are also reported.

**Discussion:**

In this study data is mainly acquired by patients' self-reporting via the internet. On-line data acquisition by patients does not require study visits to the hospital and can easily be integrated into daily life. The web-based nature of the study is believed to prevent missing data and study drop-outs. Moreover, the automated process of filling in questionnaires ensures completeness and consistency, thus improving data quality. The combination of patient-reported outcomes, fully web-based data capture and nation-wide information to all eligible patients are distinguishing features of the study and contribute to its scientific potential.

**Trial registration:**

Netherlands Trial Register (NTR): NTR2432

## Background

### Multiple sclerosis

Multiple sclerosis (MS) is a chronic inflammatory demyelinating disease of the central nervous system, for which no definite cure is available. Most patients start with a relapsing-remitting (RR) phase during which incomplete remissions often cause an increase in disability. Glatiramer acetate (GA), interferon-beta (INFb)-1a and INFb-1b are first-line disease-modifying drugs (DMDs) for RRMS treatment [1], reducing relapses and disability progression [1]. They are injected subcutaneously (GA every day; INFb-1a thrice weekly; INFb-1b every other day) or intramuscular (INFb-1a once a week). Influenza-like symptoms and depression are typical side effects of INFb, whereas pseudo-anginose symptoms immediately after injection are specific for GA. Phase 4 studies show that both INFb-1a [2] and GA treatment [3] are associated with an increase in HR-QoL. In the long term DMDs may prevent or delay increase in disability and conversion to secondary progressive MS [4].

### Adherence

Adherence to pharmacotherapy is inadequate in 30% to 50% of patients, irrespective of disease, prognosis or treatment settings [5,6]. Medication for chronic illness is only taken by 50% to 60% of patients as prescribed [7,8].

There are two levels of inadequate adherence: a) missing doses and b) discontinuation for other reasons than severe or persistent side effects or insufficient clinical benefit [6]. Data on adherence to DMDs in RRMS patients is scarce. Tremlett et al. found that 1 in 10 patients on DMD missed more than 10 doses in a 6 month period [9]. Missed doses were positively associated with alcohol consumption, and a history of missed doses predicted future missed doses [9]. The best adherence is reported in low-frequency IFNb, as compared to high-frequency IFNb or GA [10]. In MS patients with two or more DMD dispensings the mean medication possession ratio (MPR) was as low as 68% for a 24 month period [11].

After 4 months up to 11% of DMD-treated RRMS patients have discontinued treatment [12], and after 6 months figures vary from 9% to 27% [13]. However, in MS-specialised academic centres discontinuation rate was only 1.7% after 6 months and 8% after 2 years [14], suggesting that adherence may relate to certain qualitative or quantitative aspects of care. Academic MS-centres provide multidisciplinary care, coordinated by MS-nurses, whereas care may be more limited in hospitals without a special interest in MS.

Studies agree that early DMD discontinuation mostly occurs in the first 12 months [9-11]. Retrospective analysis of data on GA use in the Netherlands in the period 2003 - 2005 showed a discontinuation rate of 23.9% within the first year [personal communication by TEVA Netherlands]. In May 2006 a standard support program, Care4MS, was made available to patients starting GA, delivered by specialised nurses. In the period 2007 - 2009 discontinuation had decreased to 17% after 12 months [personal communication by TEVA Netherlands].

### Factors relating to adherence

Factors relating to the missing of doses may be different from those relating to discontinuation [9]. The WHO 2003 report on adherence puts evidence-based factors into five domains [6]: factors related to 1) the socio-economic situation, 2) health care and caregivers, 3) disease, 4) treatment, and 5) the patient. Only a few studies dealt with adherence-related factors in DMD treatment in RRMS [9]. A retrospective study in the U.S.A. among GA-treated patients identified four variables as predictors of adherence: self-efficacy, hope, perception of support, and no previous immunomodulation [15]. In a prospective study in the U.S.A. pre-treatment self-efficacy expectations were significantly related to 6-month adherence [16]. Education and optimal support of patients starting a DMD are thought to improve adherence and patient outcomes [17].

A pilot study in the Netherlands assessed 16 patient-related items that might affect GA adherence: the attitude towards self-injecting, previous immunomodulation, the reason for switching, concurrent chronic disease, disability, cognition, disease duration, type of skin, bodily constitution, working in the open, acceptance of disease, family support, social activities, personality, motivation and linguistic barrier [unpublished data]. Scoring each item for presence (1) or absence (0) the adding up of the scores yields the Discontinuation Risk Score (DRS), ranging from 0 to 16. Preliminary data suggest that patients with a DRS > 7 have an increased risk of GA discontinuation.

### Study rationale

According to the WHO 2003 report [6] and a recent Cochrane Review [18] multidisciplinary interventions pertaining to factors in all five WHO domains [6] may improve both levels of adherence. Knowledge on which qualitative or quantitative aspects of care are associated with adherence could guide the care given to patients starting a DMD. Moreover, the determination of factors prognostic of adherence would enable identification of patients at risk of inadequate adherence. As a result, adherence-promoting care could become more effective and efficient.

At present new DMDs for RRMS are becoming available. More knowledge on which aspects of multidisciplinary care are related to adherence, and the factors prognostic of inadequate adherence could increase the chances that the new efficacious drugs become effective treatments.

We conceived the idea to investigate adherence and multidisciplinary care in RRMS patients starting DMD treatment. The INFbs and GA have clearly different side effect profiles and injection frequencies. As both these aspects of treatment are likely to influence adherence we choose to study patients treated with one type of DMD, thus guaranteeing a homogenous study population. Because pilot data on discontinuation and risk factors were available for GA-treated patients in the Netherlands, it was decided to study patients starting GA treatment.

## Methods/Design

### Objectives

Appendix A lists the questions of the study.

Primary objective is to investigate whether GA adherence is associated with specific disciplines of care or quantities of specific care.

Secondary objective is to investigate whether GA adherence is associated with specific characteristics of socio-economic situation, health care or caregivers, disease, treatment, or the patient.

Tertiary objective is to assess the predictive value of the DRS with respect to GA adherence.

### Study design and treatment

An investigator-initiated, prospective, web-based, patient-centred, nation-wide, observational cohort study in the Netherlands. The study's name is an acronym from the principal analytical method, main theme and study population: Correlative analysis of Adherence In Relapsing remitting MS: CAIR. The study duration is 12 months. Nederlands Trial Register (NTR) code: TC2432.

GA is prescribed by neurologists as per regular care and dispensed as a commercial drug by general pharmacies (Copaxone^®^). GA is administered by the patient according to the instructions in the package leaflet.

### Outcomes

Primary outcomes:

a) relations between numbers of missed doses and numbers of care sessions per discipline s. quantity of care per discipline,

b) relations between number of patients who discontinued treatment and numbers of care sessions per discipline s. quantity of care per discipline.

Secondary outcomes:

a) relations between numbers of missed doses and presence s. degree of specific characteristics of the patient, socio-economic situation, health care, caregivers, disease, or treatment,

b) relations between number of patients who discontinued treatment, and presence s. degree of specific characteristics of the patient, socio-economic situation, health care, caregivers, disease, or treatment.

Tertiary outcomes:

a) predictive value of DRS with respect to missed doses,

a) predictive value of DRS with respect to number of patients who discontinued treatment.

### Patient recruitment

Patients are informed by neurologists, MS-nurses, or specialised nurses who teach patients to self-inject, and the websites of patient organisations. Patients are also advised to visit the study website (http://www.cairstudie.nl). For further information they may contact the study helpdesk by telephone or e-mail, or the coordinating investigator (PJJ) by e-mail.

Recruitment period is from July 2009 to July 2011 and comprises two phases.

#### - First phase

In 2009 15 neurological practices with a special interest in MS care and the respective MS-nurses were recruited as investigators. Practices were fairly distributed over the whole country. Since July 2009 they inform patients starting GA treatment of the possibility of participation in the study. Objectives and overall requirements are discussed with the patient. The study information is preceded by and independent from the decision to start GA. When a patient decides to participate, the neurologist or the MS-nurse notifies the helpdesk and participation is then activated.

#### - Second phase

Since February 2010 nurses who teach patients to self-inject briefly inform patients of the study. Patients interested in receiving further information are handed a postage paid card addressed to the study helpdesk. On receipt of the card the helpdesk contacts the patient by phone and gives information. Patients who, after being informed, are willing to participate either sign the informed consent form at their neurologists' or MS-nurses' office or, in case the neurologist is not yet involved in the study, confirm the text of the informed consent by clicking on a specific page on the study website. In the latter case the coordinating investigator contacts the neurologist by telephone or e-mail to introduce the study, and provides the study protocol, study synopsis and informed consent text. Within two weeks a second contact is established and the neurologist informs the coordinating investigator on his/her decision to participate or not. If the neurologist participates he/she and the MS-nurse are contacted by the helpdesk and the site is activated. A negative decision by the neurologist does not interfere with the patient's participation, as the study is patient-centred and the primary research question may be answered by patient-derived data only.

### Eligibility criteria

Eligibility criteria are minimal.

Inclusion criteria: 1) indication for GA treatment, 2) being relapse free and having stable symptoms for at least 30 days, 3) willing and able to comply with the protocol for the duration of the study, 4) having given written informed consent.

Exclusion criteria: 1) contra-indication to GA as defined in the Summary of Product Characteristics text, 2) hypersensitivity to GA or mannitol, 3) worsening of symptoms suggestive of relapse, 4) pregnancy or lactation, 5) the time interval between the first GA injection and baseline assessment is more than 4 weeks.

### Ethics

The protocol has been submitted to the Independent Review Board (IRB), an approved ethical committee residing in Amsterdam, the Netherlands. The IRB concluded that, because of the observational design of the study, a review by an ethical committee was not required, as the study did not qualify for being tested according to the Dutch Medical Research Involving Human Subjects Act of 1999 [19].

### On-line assessments and measures

Data is acquired via the study website. Patients log in with a code provided by the help desk and choose a username and password. On-line they go through various web pages containing the case record forms (CRFs) with questions and questionnaires. These electronic CRFs (eCRFs) are similar to paper questionnaires and data sheets. Questions relate to missing doses, GA discontinuation, adverse events, medication and relapses. Questionnaires pertain to self-efficacy, mood, other adherence-related factors and HR-QoL.

Patients, neurologists and MS-nurses are informed by e-mail that an assessment is due and that the corresponding eCRFs have been made available for completion. eCRFs are to be completed within one week. Within this time frame eCRFs may be filled in at moments that are suitable to the participant. Completion may take as many sessions as needed, as answers are saved automatically. After confirmation by the participant the eCRF is automatically sent to the study centre. Incomplete eCRFs are returned. In case an eCRF has not been completed within one week the help desk reminds the participant by telephone. Patients who do not have a personal computer (PC) at their disposal or who are not capable of using a PC are sent a paper-and-pencil version of the questionnaires, whereas short eCRFs are completed by telephone.

#### - Adherence

The number of missed doses in the preceding 14 days, and discontinuation of GA, the date of discontinuation and the date of restart, which ever is applicable, are recorded by patients at 3, 6, 9 and 12 months. In addition, the number of missed doses are documented at 6 random time points unknown to patients, neurologists and MS-nurses. Neurologist or MS-nurse also record the date of discontinuation or the date of restart.

#### - Received care

Care given by the following disciplines is assessed 1) neurologist, 2) MS-nurse, 3) general practitioner 4) home care 5) psychologist, 6) occupational therapy, 7) physiotherapy, 8) social worker, 9) dietician, 10) pharmacist, 11) rehabilitation doctor, 12) other medical specialists, 13) community care, 14) others. Care includes 'physical' contacts, contacts by telephone, internet, health-promoting activities that are coached or counselled by caregivers (e.g. medical fitness), or any community care activities.

Care received in the preceding 14 days is assessed by patients at baseline and bi-weekly (once every 2 weeks) thereafter up to week 52. For every discipline in which care is received, the number of care sessions and time per care session (in minutes, by approximation) are recorded. Moreover, every 3 months neurologists and nurses record the care disciplines to which they have referred the patient.

#### - Self-efficacy

Self-efficacy is the subjective belief that one can overcome challenges that one is faced with, and has been found to be a determinant of GA adherence in patients with RRMS [15]. Self-efficacy is assessed by the MS Self-Efficacy Scale (MSSES). The MSSES is a short, 14-item, psychometrically validated measure of self-efficacy specifically for use in people with MS [20]. Each item is presented with a 6-point Likert scale, ranging from 'strongly disagree' to 'strongly agree'. The MSSES score ranges from 14-84, a higher score indicating an elevated level of self-efficacy. The scale has shown sensitivity to detecting change following a therapeutic intervention.

#### - Mood and health-related quality of life

Mood is assessed by the questions 25 and 28 from the Multiple Sclerosis Quality of Life-54 questionnaire (MSQoL-54) [21]. Health-related quality of life (HR-QoL) is an overall measure of effectiveness from a patient's perspective. The MSQoL-54 is a psychometrically validated MS-specific multi-dimensional inventory of patient-centered health status. It consists of the 36-item Short Form (SF-36) health survey as a generic core measure to enable comparisons to other patient populations and to the general population, supplemented with 18 additional questions exploring items relevant to patients with MS [21]. A physical and a mental dimension underlie the MSQoL-54 [21]. Scores for the physical and the mental domain range from 0 to 100, where higher values indicate better HR-QoL.

#### - Adherence-related factors

The Dutch Adherence Questionnaire-90 (DAQ-90) is a 90-item questionnaire designed to assess all factors identified by the WHO 2003 report [6] as evidence-based determinants of adherence (Appendix B). The DAQ-90 consists of 5 sections corresponding to the five domains of adherence-related factors in the WHO 2003 report [6]. The DAQ-90 includes questions on hope and the perception of support and is completed by the patient at the end of the study.

#### - Discontinuation Risk Score

At baseline the 16 items of the DRS are scored for absence (0) or presence (1) by the nurses who teach patients to self-administer. The adding up of item scores yields the DRS.

#### - Adverse events

Adverse events considered by patients, neurologists or nurses as probably or definitely related to GA treatment are reported on-line at 3, 6, 9 and 12 months. As the study is observational and does not interfere with care, patients always report adverse events to their neurologist or MS-nurse. Serious adverse events are recorded by neurologists or MS-nurses and reported to the coordinating investigator within 48 hours.

#### - Disease characteristics

At baseline neurologists or MS-nurses provide the following data: disease course, disease duration, medication, previous immunomodulation, the number of relapses in the last 12 and 24 months, the number of steroid-treated relapses in the last 12 and 24 months. Assessment of disability (Expanded Disability Status Scale [EDSS] score) at baseline and 12 months is optional. Relapses occurring during the study and eventual steroid treatment are reported by patients and neurologists or MS-nurses at months 3, 6, 9 and 12.

### Schedule of assessments

The schedule of assessments is presented in Table 1.

**Table 1:** Schedule of assessments.

	Baseline	Month 3	Month 6	Month 9	Month 12
Adherence*	X	X	X	X	X

MSSES	X				X

MSQoL-54	X		X		X

Adverse Events	X	X	X	X	X

Relapses	X	X	X	X	X

EDSS**	X				X

DAQ-90					X

DRS	X				

*, and at 6 additional random time points

**, optional

MSSES, Multiple Sclerosis Self Efficacy Scale

MSQoL-54, Multiple Sclerosis Quality of Life-54

EDSS, Expanded Disability Status Scale

DAQ-90, Dutch Adherence Questionnaire-90

DRS, Discontinuation Risk Score

### Power calculation

In order to determine the size of the study, we first assumed that the analysis would be based on a t-test: For each factor we form three tertiles: patients with low, intermediate and high scores. When a t-test is carried out to compare a parameter between the lowest and highest tertile, 65 patients per tertile are required in order to detect a difference of 0.5 standard deviations with 80% power (two-sided alpha = 0.05). As we will use linear regression instead of a t-test, the power of the regression analysis may even be higher, but will be at least comparable. In a previous on-line study in GA-treated patients compliance was very high, both with respect to missing data and drop outs [22]. Therefore a total of 200 patients will need to be enrolled.

In phase II of the recruitment all Dutch patients who start GA treatment are informed of the study. Based on data from a previous study we hypothesize that 50% of patients are willing to participate [22]. Based on recent prescription data it is expected that approximately 300 patients start GA treatment in the Netherlands per year. So, it is calculated that in phase II recruitment is 150 per year, and that recruitment will end Q2 2011. As per October 2010, 119 patients have been enrolled.

### Statistical analyses

Per discipline the relations between missed doses and the number of care sessions s. care duration will be evaluated using linear regression; and the relations between discontinuation and the number of care sessions s. care duration will be evaluated in a similar way, but by a proportional hazard analysis, with stepwise selection. In addition, univariable analyses will be carried out. Two-sided 95% confidence intervals will be calculated.

### Reporting of results

A manuscript, based on the study report, will be submitted to a peer-reviewed international scientific journal for publication. Oral or poster communications will be given on international and national symposia. Participating patients will receive a summary of the study report (findings, conclusions), written in a wording that is understandable to lay persons, as soon as the report has been finalized.

## Discussion

The CAIR study investigates relations between adherence to GA treatment and disciplines or quantity of care, based on data on missing doses, discontinuation and received care as reported by patients. Neurologists or MS-nurses may refer patients to caregivers or give advice on health promoting activities. However, as adherence is often sub-optimal in patients with chronic disease, self-reporting by patients on the care they actually did receive is likely to better reflect reality [23].

The patient-centred character of the study is realized by the web-based approach. Data is obtained by the patient's on-line self-reporting. Thus, data acquisition does not require study visits to the hospital and can be integrated into daily life. This feature of the study is believed to promote compliance and prevent study drop-outs. Moreover, the use of the internet contributes to data quality assurance, as the automated process of data capture ensures completeness and consistency.

According to the cohort-based, nation-wide design we aim to inform every RRMS patient in the Netherlands who starts GA treatment. Neurologists and MS-nurses in 15 MS-dedicated hospitals, evenly distributed over the country, actively recruit patients. In addition, the nurses, who teach patients to self-inject, inform patients of the possibility of participating. Patients' awareness of the study is promoted by information on websites from national MS patients organisations. Recruitment is on schedule, with 119 patients being enrolled as per October 2010.

In all, the combination of patient-reported outcomes, fully on-line data capture, and nation-wide information of all eligible patients are distinguishing features of the CAIR study. The nation-wide cohort design makes sure that results will be applicable to Dutch RRMS patients. Implementation of the study's conclusions might well improve the effectiveness of present and future DMD treatments, and increase the efficiency of MS care.

## Competing interests

Dr. Jongen has received honoraria from Merck-Serono, Novartis, Bayer-Schering, Biogen-Idec, Sanofi-Aventis, Teva, and Allergan for activities as speaker or advisory committee member, research support, or travel grants for conferences.

## Authors' contributions

PJJ initiated the study, contributed to the conception and design of the study, coordinates the study, and drafted the manuscript. GH contributed to the conception and design of the study. RH, HS, JG, JHV, EH, MvH, EvM, JS, EdS, TS, MT, EZ, JtH and RvdK all contributed to the acquisition of data. GB contributed to the conception and design of the study and co-drafted the manuscript. All authors read and approved the final version of the manuscript.

## Authors' information

Peter Joseph Jongen is a neurologist and the founding director of the MS4 Research Institute, Nijmegen, the Netherlands. He has been involved in MS clinical research and patient care for more than 15 years. He is a member of the International Medical and Scientific Board of the Multiple Sclerosis International Federation (MSIF), a former director of the MS Centre Nijmegen, a former council member of the European Committee for Treatment and Research in Multiple Sclerosis (ECTRIMS), and author of over 90 peer-reviewed scientific articles. The MS4 Research Institute is an independent non-profit organisation, that conceives, performs, and coordinates scientific research on the therapeutic value of treatments in MS.

## Appendix A

List of Questions of the Study

I. What are the relations between GA adherence and multidisciplinary care?

1. Is the quantity of care given by the *neurologist *(number of contacts, total duration of contacts) related to GA adherence (missed doses, discontinuation)?

2. Is (the quantity of) care given by the *MS-nurse *(number of contacts, total duration of contacts) related to GA adherence (missed dosed, discontinuation)?

3. Is (the quantity of care) given by the *general practitioner *(number of contacts, total duration of contacts) related to GA adherence (missed doses, discontinuation)?

4. Is (the quantity of) *home care *(number of contacts, total duration of contacts) related to GA adherence (missed doses, discontinuation)?

5. Is (the quantity of) care given by a *psychologist *(number of contacts, total duration of contacts) related to GA adherence (missed doses, discontinuation)?

6. Is (the quantity of) *occupational care *(number of contacts, total duration of contacts) related to GA adherence (missed doses, discontinuation)?

7. Is (the quantity of) *physiotherapy *(number of contacts, total duration of contacts) related to GA adherence (missed doses, discontinuation)?

8. Is (the quantity of) care given by a *social worker *(number of contacts, total duration of contacts) related to GA adherence (missed doses, discontinuation)?

9. Is (the quantity of) care given by a *dietician *(number of contacts, total duration of contacts) related to GA adherence (missed doses, discontinuation)?

10. Is (the quantity of) care given by the *pharmacist *(number of contacts, total duration of contacts) related to GA adherence (missed doses, discontinuation)?

11. Is (the quantity of) care given by a *rehabilitation doctor *(number of contacts, total duration of contacts) related to GA adherence (missed doses, discontinuation)?

12. Is (the quantity of) *community care *(number of contacts, total duration of contacts) related to GA adherence (missed doses, discontinuation)?

II. **What characteristics of the patient, the socio-economic situation, health care or caregivers, disease, or treatment are related to GA adherence?**

1. Is self-efficacy related to GA adherence?

2. Is mood related to GA adherence?

3. What other characteristics of the patient are related to GA adherence?

4. What characteristics of the socio-economic situation, health care or caregivers, disease, or treatment are related to GA adherence?

III. What is the predictive value of the DRS with respect to GA adherence?

1. What is the predictive value of the DRS with respect to GA discontinuation?

2. What is the predictive value of the DRS with respect to missed GA doses?

## Appendix B

Dutch Adherence Questionnaire-90 (DAQ-90) (Table 2)

Based on WHO report 2003

^© ^P.J. Jongen, MS4 Research Institute, Nijmegen, the Netherlands 2009

Version for Glatiramer Acetate

Patient ID: ........................................................................

Date: ..............................................................................

Study site: ........................................................................

**Table 2:** Dutch Adherence Questionnaire-90 (DAQ-90)

**I. Algemene/socio-economische omstandigheden**	
1. Bent u tevreden over uw economische situatie?	JA/NEE
2. Vindt u uw besteedbaar maandinkomen voldoende?	JA/NEE
3. Bent u voldoende vaardig in schrijven en lezen?	JA/NEE
4. Wat is de hoogste afgemaakte opleiding?	..............
5. Was u in het afgelopen jaar (tijdelijk) werkeloos?	JA/NEE
6. Had u in het afgelopen jaar deeltijdwerk?	JA/NEE
7. Ervaart u voldoende steun van uw sociale netwerken?	JA/NEE
8. Zijn uw leefomstandigheden stabiel?	JA/NEE
9. Hoever is de afstand tot MS polikliniek/behandelcentrum?	... km
10. Heeft u een eigen bijdrage aan vervoerskosten van - naar polikliniek/behandelcentrum?	JA/NEE
11. Heeft u een eigen bijdrage aan medicijnkosten?	JA/NEE
12. Is het afgelopen jaar uw leefomgeving veranderd?	JA/NEE
13. Waren er het afgelopen jaar gezinsproblemen?	JA/NEE/n.v.t.
**II. Gezondheidszorg**	
14. Hoelang duurde het eerste neurologisch consult op deze polikliniek/in dit behandelcentrum?	.... min.
15. Hoelang duren gemiddeld de neurologische vervolgconsulten?	.... min.
16. Hoelang duurde het eerste MS-verpleegkundig consult op deze polikliniek/in dit behandelcentrum?	.... min.
17. Hoelang duren gemiddeld de MS-verpleegkundige vervolgconsulten?	.... min.
18. Vindt u de continuïteit van uw MS-zorg voldoende?	JA/NEE
19. Is meestal dezelfde neuroloog voor u beschikbaar?	JA/NEE
20. Is meestal dezelfde MS-verpleegkundige voor u beschikbaar?	JA/NEE
21. Bent u tevreden over de communicatie met uw neuroloog?	JA/NEE
22. Bent u tevreden over de communicatie met uw MS-verpleegkundige?	JA/NEE
23. Heeft u een goede relatie met uw neuroloog?	JA/NEE
24. Heeft u een goede relatie met uw MS-verpleegkundige?	JA/NEE
25. Vindt u de kwaliteit van de Nederlandse gezondheidszorg goed ?	JA/NEE
26. Worden uw behandelingen en medicijnen volledig vergoed?	JA/NEE
27. Wordt Copaxone altijd op tijd geleverd?	JA/NEE
28. Hebben de zorgverleners voldoende kennis van MS?	JA/NEE
29. Zijn de zorgverleners regelmatig overwerkt?	JA/NEE
30. Bent u door de zorgverleners voldoende geïnformeerd over MS?	JA/NEE
31. Is met u voldoende besproken hoe u klachten zelf gunstig kunt beïnvloeden?	JA/NEE
32. Weet u wat adherentie is?	JA/NEE
33. Vindt u adherentie belangrijk?	JA/NEE
**III. Ziektegerelateerde factoren**	
34. Heeft u veel last van MS?	JA/NEE
35. Heeft u veel beperkingen t.g.v. MS?	JA/NEE
36. Is uw MS het afgelopen jaar erg actief geweest?	JA/NEE
37. Zijn er volgens u effectieve behandelingen van MS?	JA/NEE
38. Kunt u inschatten wat het hebben van MS voor u op termijn kan betekenen?	JA/NEE
39. Vindt u Copaxone een adequate behandeling voor uw MS?	JA/NEE
40. Heeft u last van depressiviteit?	JA/NEE
41. Heeft u andere ziekten behalve MS?	JA/NEE
42. Kunt u niet zonder bepaalde geneesmiddelen of bent u afhankelijkheid van bepaalde geneesmiddelen?	JA/NEE
43. Bent u verslaafd aan drugs?	JA/NEE
44. Bent u afhankelijk van of verslaafd aan alcohol?	JA/NEE
45. Hoeveel eenheden alcoholhoudende drank drinkt u per week?	per week
**IV. Behandelinggerelateerde factoren**	
46. Indien u behalve Copaxone nog andere medicijnen gebruikt: Heeft u een ingewikkeld schema om alle medicijnen in te nemen?	JA/NEE/n.v.t.
47. Vindt u behandeling met Copaxone te lang duren?	JA/NEE
48. Bent u eerder met interferon gestopt wegens onvoldoende werkzaamheid?	JA/NEE
49. Bent u eerder met interferon gestopt wegens bijwerkingen?	JA/NEE
50. Heeft u voorafgaande aan Copaxone twee of meer andere middelen gebruikt om terugvallen te voorkomen?	JA/NEE
51. Verwachtte u binnen enkele weken gunstige effecten van Copaxone?	JA/NEE
52. Ondervond u voldoende ondersteuning om met bijwerkingen om te gaan?	JA/NEE
**V. Patiëntgerelateerde factoren**	
53. Vindt u uw kennis en opvattingen over MS adequaat?	JA/NEE
54. Bent u gemotiveerd om zelf uw MS te 'managen'?	JA/NEE
55. Heeft u er vertrouwen in dat u uw gedrag/gewoontes kunt veranderen?	JA/NEE
56. Heeft u reële verwachtingen t.a.v. het resultaat van Copaxone behandeling?	JA/NEE
57. Weet u wat het missen van injecties Copaxone voor u kan betekenen?	JA/NEE
58. Heeft u last van vergeetachtigheid?	JA/NEE
59. Heeft u last van stress?	JA/NEE
60. Bent u bang voor bijwerkingen van Copaxone?	JA/NEE
61. Was u bang voor bijwerkingen toen u met Copaxone startte?	JA/NEE
62. Heeft u weinig motivatie om Copaxone te spuiten?	JA/NEE
63. Had u weinig motivatie toen u met Copaxone startte?	JA/NEE
64. Kunt u goed met uw MS klachten omgaan?	JA/NEE
65. Kunt u goed uw behandeling zelf 'managen'?	JA/NEE
66. Vindt u de Copaxone behandeling nodig?	JA/NEE
67. Merkt u dat Copaxone behandeling effect heeft?	JA/NEE
68. Bent u negatief over de werkzaamheid Copaxone?	JA/NEE
69. Heeft u MS geaccepteerd?	JA/NEE
70. Twijfelt u aan de diagnose MS?	JA/NEE
71. Twijfelde u aan de diagnose MS toen u met Copaxone startte?	JA/NEE
72. Bent u op de hoogte van de mogelijke risico's van MS voor uw gezondheid?	JA/NEE
73. Bleek achteraf dat u instructies over Copaxone behandeling verkeerd had begrepen?	JA/NEE
74. Mist u wel eens een controleafspraak?	JA/NEE
75. Heeft u geringe verwachtingen van Copaxone behandeling?	JA/NEE
76. Heeft u last van hopeloosheid of negatieve gevoelens?	JA/NEE
77. Bent u gefrustreerd over bepaalde zorgverlener(s)?	JA/NEE
78. Bent u bang afhankelijk te zijn?	JA/NEE
79. Was u bang dat Copaxone uw MS behandeling te ingewikkeld zou maken?	JA/NEE
80. Heeft u het gevoel dat u door MS een bepaald stempel heeft gekregen?	JA/NEE
81. Was u bezorgd door Copaxone plots uw leefstijl te moeten veranderen?	JA/NEE
82. Maakt u zich zorgen over de effecten van Copaxone op lange termijn?	JA/NEE
83. Bent u in het algemeen negatief over het gebruik van medicijnen?	JA/NEE
84. Vindt u dat artsen in het algemeen teveel medicijnen voorschrijven?	JA/NEE
85. Bent u in het algemeen argwanend jegens chemische stoffen in voedsel en milieu?	JA/NEE
86. Bent u in het algemeen argwanend jegens wetenschap of technologie?	JA/NEE
87. Vindt u het belangrijk dat u met Copaxone wordt behandeld?	JA/NEE
88. Had u er bij start van Copaxone vertrouwen in de behandeling te kunnen uitvoeren?	JA/NEE
89. Bent u vaak op reis?	JA/NEE
90. Heeft u onregelmatige werktijden?	JA/NEE

^© ^P.J. Jongen, MS4 Research Institute, Nijmegen, the Netherlands 2009

## Pre-publication history

The pre-publication history for this paper can be accessed here:

http://www.biomedcentral.com/1471-2377/11/40/prepub
